# Patient Journey Toward a Diagnosis of Light Chain Amyloidosis in a National Sample: Cross-Sectional Web-Based Study

**DOI:** 10.2196/44420

**Published:** 2023-11-02

**Authors:** Xuelin Dou, Yang Liu, Aijun Liao, Yuping Zhong, Rong Fu, Lihong Liu, Canchan Cui, Xiaohong Wang, Jin Lu

**Affiliations:** 1 Hematology Department Peking University People's Hospital Beijing China; 2 Hematology Department Shengjing Hospital of China Medical University Shenyang China; 3 Hematology Department Qingdao Municipal Hospital Qingdao University Qingdao China; 4 Hematology Department Tianjin Medical University General Hospital Tianjin China; 5 Hematology Department The Fourth Hospital of Hebei Medical University Shijiazhuang China; 6 Medical Affairs Xi’an Janssen Pharmaceutical Ltd Beijing China; 7 Medical Affairs Xi’an Janssen Pharmaceutical Ltd Shanghai China

**Keywords:** systemic light chain amyloidosis, AL amyloidosis, rare disease, big data, network analysis, machine model, natural language processing, web-based

## Abstract

**Background:**

Systemic light chain (AL) amyloidosis is a rare and multisystem disease associated with increased morbidity and a poor prognosis. Delayed diagnoses are common due to the heterogeneity of the symptoms. However, real-world insights from Chinese patients with AL amyloidosis have not been investigated.

**Objective:**

This study aimed to describe the journey to an AL amyloidosis diagnosis and to build an in-depth understanding of the diagnostic process from the perspective of both clinicians and patients to obtain a correct and timely diagnosis.

**Methods:**

Publicly available disease-related content from social media platforms between January 2008 and April 2021 was searched. After performing data collection steps with a machine model, a series of disease-related posts were extracted. Natural language processing was used to identify the relevance of variables, followed by further manual evaluation and analysis.

**Results:**

A total of 2204 valid posts related to AL amyloidosis were included in this study, of which 1968 were posted on haodf.com. Of these posts, 1284 were posted by men (median age 57, IQR 46-67 years); 1459 posts mentioned renal-related symptoms, followed by heart (n=833), liver (n=491), and stomach (n=368) symptoms. Furthermore, 1502 posts mentioned symptoms related to 2 or more organs. Symptoms for AL amyloidosis most frequently mentioned by suspected patients were nonspecific weakness (n=252), edema (n=196), hypertrophy (n=168), and swelling (n=140). Multiple physician visits were common, and nephrologists (n=265) and hematologists (n=214) were the most frequently visited specialists by suspected patients for initial consultation. Additionally, interhospital referrals were also commonly seen, centralizing in tertiary hospitals.

**Conclusions:**

Chinese patients with AL amyloidosis experienced referrals during their journey toward accurate diagnosis. Increasing awareness of the disease and early referral to a specialized center with expertise may reduce delayed diagnosis and improve patient management.

## Introduction

AL amyloidosis is a rare and multisystem disease with an incidence of 3 to 14 persons per million per year [1]. The median age at diagnosis is 63 years [2]. The disease occurs from the misfolding of proteins that become deposited in organs (heart, kidney, liver, soft tissues, peripheral nervous system, and gastrointestinal tract), leading to progressive organ damage and impairment of quality of life [3]. Unfortunately, for most patients the disease goes unrecognized until severe organ dysfunction develops. Establishing a diagnosis of amyloidosis is difficult, and 37.1% of respondents in a patient experience survey said that diagnosis was not established until more than one year after the onset of initial symptoms [4]. The prognosis of patients with AL amyloidosis is poor, with only 20% of patients surviving over 10 years [5]. The Mayo Clinic reported that earlier diagnosis led to fewer patients who died within 6 months of diagnosis in the most recent period [6].

Nevertheless, delayed diagnosis is often seen in AL amyloidosis because of the low specificity of the phenotype and early symptoms and a complex, multistage diagnostic process [7]. The median time from symptom to diagnosis was 7 months in a Chinese hospital, and 37% of patients were diagnosed with stage III disease by the Mayo 2004 staging [8]. The prognosis of Chinese patients with AL amyloidosis is poor, with a median survival time of 36.3 months, and one-third of patients die within one year [9]. Once cardiac involvement is present, inferior overall survival accelerates, underlining the need for early referral and diagnosis [10].

There is a lack of evidence to describe the experience of patients with AL amyloidosis when they are diagnosed. To obtain an overall understanding of the journey to diagnosis, conventional methods include structured in-depth interviews, questionnaires, or real-world research [11]. The emergence of social media platforms has significantly changed the means of communication, and social listening is a novel strategy for understanding users’ needs [12,13]. Social listening is applied to collect data based on specific themes or time frames and to analyze the data for a certain purpose. It provides complementary perspectives for data collection and data analysis, especially when data are unable to be widely collected in real life. This technique has been used to describe symptoms, patient experience, and communications between doctors and patients in diverse diseases, such as Parkinson disease, COVID-19, dry-eye diseases, and inflammatory bowel diseases [12,14-17]. In China, online consultations can serve as an effective communication tool in physician-patient relationships and have already acted as a supplementary tool for health care, with 88,308 doctors in China registered to provide online consultation as of September 23, 2017 [18], thus making it possible for our study to collect data from online consultations as a source of real-world clinical practice and use machine learning to obtain insights on disease characteristics, patient referrals, and diagnoses and treatments. This will provide a reference for optimizing the patient journey for AL amyloidosis in China.

## Methods

### Study Design and Data Source

The objective of this study was to describe the journey of Chinese patients with AL amyloidosis from symptom onset through to diagnosis. A systematic search was conducted using the predefined search terms *amyloid* and *amyloidosis* in both Chinese and English from online medical consultation platforms in mainland China (haodf.com, wy.guahao.com, chunyuyisheng.com, ask.39.net and 91160.com) between January 2008 and April 2021.

### Ethical Considerations

All data used and presented in this study were obtained from publicly accessible sources without accessing password-protected information and did not contain personal or sensitive data. Ethics approval was not required due to the public nature of the internet-based data used. All online content was anonymized in compliance with Chinese data privacy obligations. No personal identifiers were collected. We conducted social listening that complied with ethical considerations based on guidance in China. Confidentiality was maintained throughout the entire project cycle from data collection to reporting.

### Post Collection and Cleaning

Web crawlers [19] were designed to extract posts with the keywords *amyloid* and *amyloidosis* in Chinese and English; 9330 posts were initially collected. A smaller sample of 7466 posts was acquired from the 9330 posts by manually excluding posts that were (1) not a complete consultation, such as parts of health care articles or concerns of a suspected patient without a doctor’s reply and (2) content that could not be traced to its source due to fee-for-service security in some online medical consultation platforms. Finally, 2204 posts were included in this study.

**Figure 1:**
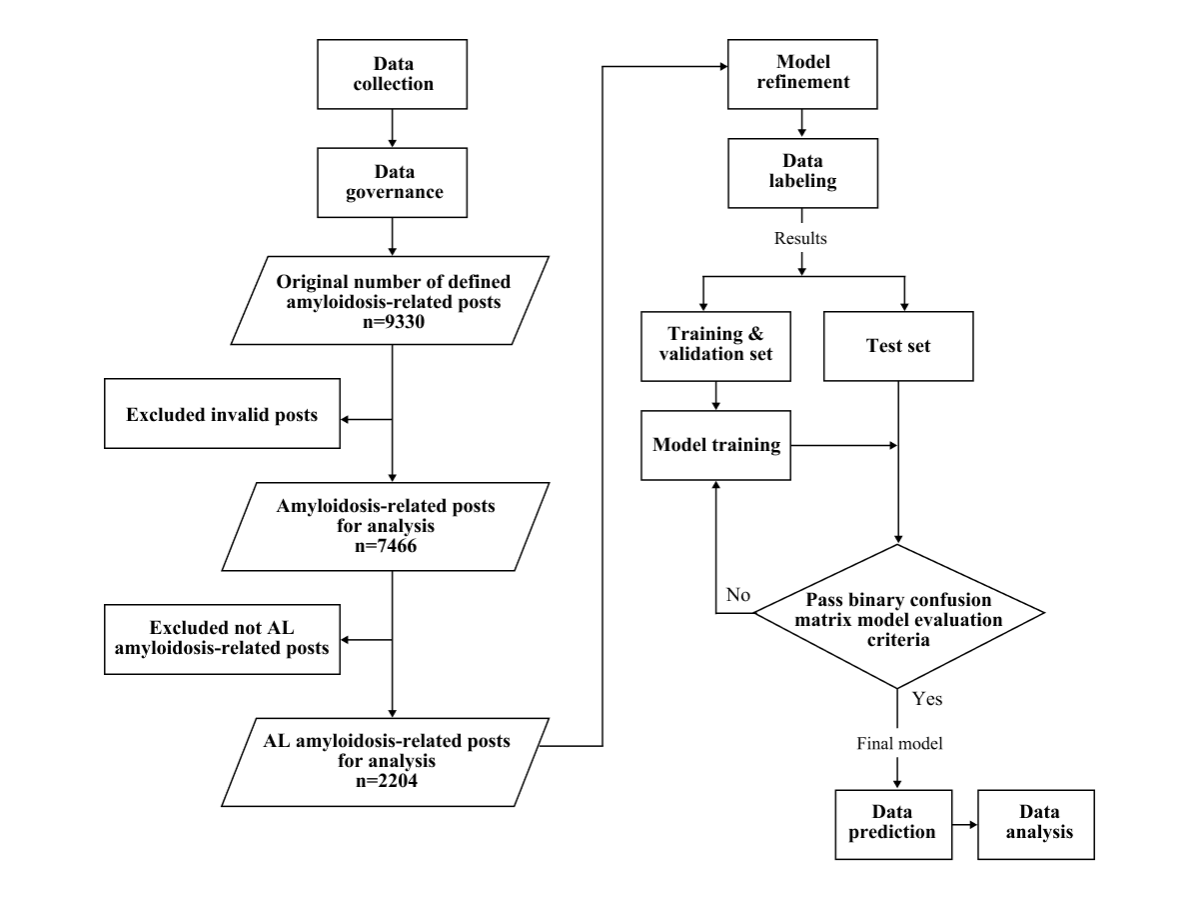
Flow chart of post selection and processing of information. The left side shows the method for data cleaning, and the right side shows the method for refinement of the natural language processing model.

Figure 1 shows the process for data cleaning from raw data to 2204 valid posts (on the left) and the method for refinement of the natural language processing (NLP) model (on the right).

### Categorization and Analysis

Manual labeling was implemented for the raw data to optimize the machine learning model. We used 500 valid posts for model refinement instead of all posts to avoid overfitting during machine learning. From the 500 posts, 80% of the labeling results were used as the training set for the model training process, 10% were used as the validation set for model optimization, and the other 10% were used as the test set to evaluate the model in a binary confusion matrix [19]. When all criteria in the matrix were passed, the model was finalized and used for data prediction of all 2024 valid posts, including named entity recognition, relation extraction, and sentiment analysis.

### NLP Model Training and Data Prediction Process

Data transformation was processed with NLP, specifically, a bidirectional encoder representations from transformers (BERT) plus conditional random field (CRF) model, which is an encoder-decoder based codec model proposed by Google. BERT is a language representation model introduced by Google AI Language designed to pretrain deep bidirectional representations from unlabeled text by jointly conditioning on both left and right context in all layers [20]. Combined with a CRF model [21], a popular structured prediction method of classification and graphical modeling, we used named entity recognition and analyzed the sentences for content. The specific content fields for entity recognition included demographic attributes (age, gender, geographical distribution); the organ involved; presentation, examination, and diagnosis; treatment; and follow-up. Next, we used pipelines for entity and relation extraction to parse out the relationships between different entity types from natural text, followed by BERT for sentiment analysis [22].

### Data Analysis Process

Descriptive summary statistics were calculated for available demographic and clinical data, and the driving factors and concepts at each key step in the disease diagnosis and treatment journey were deeply explored with machine learning [23]. According to the data mentioned above (Figure 1), we could match these specific data to an identical suspected patient through the source of information, temporal distribution, geographical distribution, and the clinical data. Content from doctors or suspected patients was also annotated and distinguished.

## Results

### Study Participants

There were 9330 valid consultations and inquiries between January 2008 and April 2021 in total. A total of 2204 posts by suspected patients with AL amyloidosis were included after excluding incorrect or incomplete posts and content not related to AL amyloidosis; 89.29% (n=1968) of these posts were from haodf.com, followed by wy.guahao.com and chunyuyisheng.com (Multimedia Appendix 1, Table S1). The number of online consultations for AL amyloidosis increased every year, reaching 461 in 2020. Geographical distribution showed that Jiangsu (n=260), Zhejiang (n=181), Beijing (n=142), Henan (n=142), Shandong (n=138), Anhui (n=131), Hebei (n=125), Hubei (n=125), Liaoning (n=118 ), and Guangdong (n=115) provinces accounted for 67.01% (n=1477) of the 2204 posts (Multimedia Appendix 1, Table S1).

A total of 1284 posts were made by male users. In 196 posts with valid data for age, the median age was 57 (IQR 46-67) years, ranging from 21 to 87 years. In the pool of 2204 posts, we collected specific symptom-related keywords to evaluate organ involvement in suspected patients with AL amyloidosis. We found that 1459 posts mentioned keywords for renal-related symptoms, followed by heart (n=833), liver (n=491), stomach (n=368), lung (n=306), intestinal (n=280), and nervous system (n=225) symptoms. Furthermore, 1502 suspected patients had 2 or more affected organs (Multimedia Appendix 1, Table S2).

### Journey to Diagnosis

**Figure 2:**
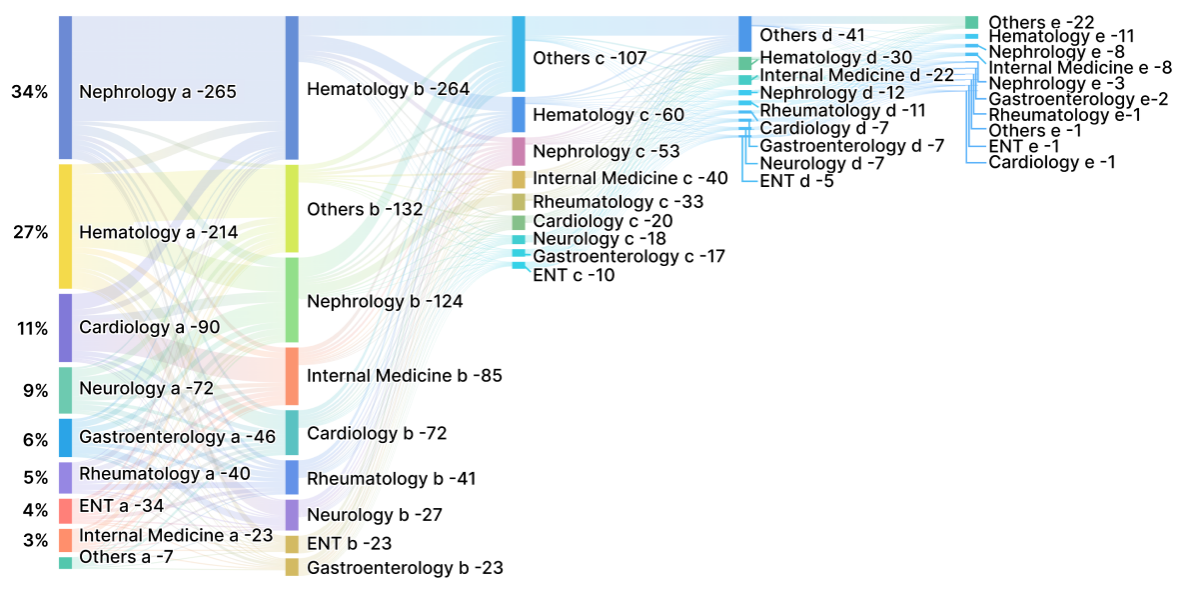
Intrahospital referrals of suspected patients with light chain amyloidosis (n=791) shown as a Sankey diagram in which the width of the arrows is proportional to the flow quantity. The source node (words labeled with "a") is the initially visited department, and the target nodes are the second (words labeled with "b") or later (words labeled with "c," "d," or "e") visited departments; the value to set the flow volume is the ratio of patients who visited the specific department versus all patients for whom department visit data were available. The labels show the department of the patients’ medical visits. ENT: ear, nose, and throat.

**Figure 3:**
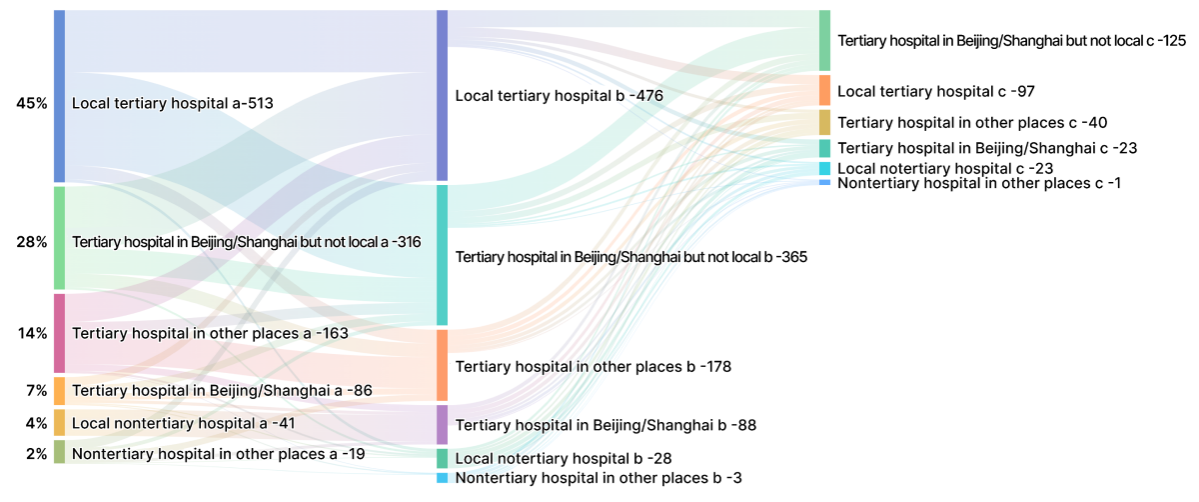
Interhospital referrals of suspected patients with light chain amyloidosis (n=1138) shown as a Sankey diagram in which the width of the arrows is proportional to the flow quantity. The source node (words labeled with "a") is the initially visited location, and the target nodes are the second (words labeled with "b") or later (words labeled with "c," "d," or "e") visited location; the value to set the flow volume is the ratio of patients who visited the specific location versus all patients for whom location data were available. The labels show the location of the patients’ medical visits. ENT: ear, nose, and throat.

Symptoms varied from patient to patient, and the interval from symptoms to diagnosis for patients with AL amyloidosis was long. Among 2204 posts, weakness, edema, and hypertrophy were the most frequently mentioned symptoms (Table 1). Among 13 posts that mentioned the time from symptom to diagnosis, eight posts reported that they took more than one year to be correctly diagnosed. In many posts, suspected patients reported that they did not consider that the symptoms were related to hematologic disorder when the symptoms began, and they took medicine without a prescription to relieve the discomfort. When the symptoms worsened, it was also difficult to be diagnosed because of the nonspecificity of the symptoms, resulting in frequent intra- and interhospital referrals. A total of 791 posts mentioned the content of the intrahospital referral (Figure 2). Multiple physician visits were reported in posts prior to diagnosis for AL amyloidosis, including to nephrologists, hematologists, cardiologists or neurologists, gastroenterologists, rheumatologists, internists, hepatologists, and otorhinolaryngologists. The first doctor seen was usually a nephrologist (n=265), hematologist (n=214), or cardiologist (n=90). The suspected patients were then often referred for a second visit to a hematologist (n=264), nephrologist (n=124), or cardiologist (n=72). As for interhospital referrals (Figure 3), suspected patients were more likely to go to tertiary hospitals. Local tertiary hospitals (n=513), tertiary hospitals in Beijing and Shanghai (n=316), and tertiary hospitals in cities other than Beijing and Shanghai (n=163) were the most popular choices. Providing an overview of interhospital referrals for suspected patients with AL amyloidosis is complicated; normally, suspected patients are referred between different tertiary hospitals in various cities, except for Beijing and Shanghai residents. Reasons reported in the posts for interhospital referrals included seeking an accurate diagnosis or seeking more effective treatment.

Although AL amyloidosis remains an incurable disease, much progress has been made in the last decade, and important aspects of clinical care for AL amyloidosis patients are guided by evidence-based treatment. Among 1688 posts by doctors mentioning treatment-related keywords, there was an increase in recommendations for bortezomib for the treatment of AL amyloidosis, whereas there was a decline in recommendations for melphalan (Multimedia Appendix 1, Figure S1A). Among 164 posts in which treatment type could be found, bortezomib was recommend as a first-line and second-line treatment by 70% (n=115) and 53% (n=87) of physicians, respectively (Multimedia Appendix 1, Figure S1B).

**Table 1:** Symptoms of suspected patients with light chain amyloidosis in China extracted from 2204 posts. Symptoms mentioned by suspected patients were categorized according to an in-house dictionary. Posts could include more than one symptom.

Symptoms	Incidence, n (%)
Weakness	252 (9)
Edema	196 (7)
Hypertrophy	168 (6)
Swelling	140 (5)
Infection	139 (5)
Proteinuria	112 (4)
Diarrhea	110 (4)
Nausea and vomiting	86 (3)
Dyspnea	85 (3)
Low fever	85 (3)
Flu-like symptoms	58 (2)
Fluid accumulation	58 (2)
Hypotension	57 (2)
Inflammation	56 (2)
Acroanesthesia	56 (2)
Wheezing	55 (2)
Poor appetite	55 (2)
Pleural fluid	31 (1)
Ostealgia	31 (1)
Abdominal distention	30 (1)
Neuroticism	30 (1)
Insomnia	29 (1)
Constipation	28 (1)
Ascites	28 (1)
Skin rash	28 (1)
Palpitation	27 (1)
Phlegm	27 (1)
Polyps	26 (1)
Foamy urine	26 (1)

## Discussion

### Principal Findings

This is the first study to provide insights into real-world consultation with Chinese patients with AL amyloidosis using a big data technique. We investigated patients’ journeys to an AL amyloidosis diagnosis over more than 10 years, including disease characteristics, patient referrals, diagnoses, and treatments. The main findings of the study are that multiple physician visits and delayed diagnosis were common among patients with AL amyloidosis in the real word.

### Comparison With Prior Work

By comparison to traditional methods based on physician and patient surveys, social listening can collect a large amount of data and provide a comprehensive understanding of real-world settings. The last 5 years have seen a leap in the development of health information technology and social media in China [13]. With 5.25% (88,308/1,680,062) of all doctors in China registered to provide online consultation on haodf.com as of September 23, 2017, online doctors had an average workload of 0.38 patients per doctor per day [18]. The emergence of online consultations provides Chinese patients with a variety of different services, including health problem discussion, health knowledge sharing and searching, diagnosis and treatment, and making appointments and registering [24]. In this study, we included 2204 posts related to AL amyloidosis from online consultations over 13 years (2009-2021). Compared to a systematic review that examined a similar period, our study reflects real-world insights from patients and has built a detailed image of intra- and interhospital referrals. Similarly, the most involved organ was the kidney in Chinese patients with AL amyloidosis, probably because Chinese nephrologists may have more experience of this rare disease than other departments; most papers on Chinese patients with AL amyloidosis are from the renal or hematology departments [25]. Only 38% (n=833) of posts mentioned cardiac-related symptoms in this study, even though cardiac involvement is more common in US patients [26]. Cardiac symptoms may be mistakenly attributed to other cardiac diseases and significantly underdiagnosed [27]. Posts from consultations generated by suspected patients might not differentiate AL amyloidosis–related cardiac symptoms from other cardiac diseases, especially among older posters. An alternative explanation is that posts that were not recognized as describing cardiac symptoms were probably made because the suspected patient or the physician did not ask about or comment on cardiac symptoms. Moreover, whether Chinese patients with AL amyloidosis have a greater susceptibility to renal involvement needs further research.

Weakness, edema, and hypertrophy were the most frequently mentioned symptoms. Likewise, a longitudinal study of community-based patients with AL amyloidosis in the United States found that nonspecific symptoms, such as fatigue and loss of appetite, were reported by 80% of patients [11]. Huang et al [9] reported that Chinese patients primarily presented with fatigue (40%) and edema (81%) at the time of diagnosis, followed by orthostatic hypotension (30%) and weight loss (27%), while in US health care institutions, the most common signs and symptoms are malaise or fatigue (61%) and dyspnea (59%) [26].

Our analysis indicates that patients experienced long journeys, visiting different departments and different hospitals until being correctly diagnosed; thus, delayed diagnoses were commonly seen in patients with AL amyloidosis. McCausland et al [11] conducted a longitudinal, noninterventional study of community-based patients with AL amyloidosis. They found that nearly 43% of patient interviewees needed more than 12 months from the onset of symptoms to a confirmed diagnosis, and 43% of patients had consulted 5 or more doctors before diagnosis [11]. In our study, 8 of 13 patients declared that they were diagnosed before 1 year after the onset of symptoms. Reflecting our results as well as those of the survey by McCausland et al [11], patients’ interpretation of their initial symptoms, timing of seeking medical help, and challenges in differential diagnoses might be barriers to diagnosis for AL amyloidosis. Symptoms of AL amyloidosis are often misinterpreted as aging or other more common chronic ailments; thus, more attention should be paid to potential AL amyloidosis–related signs and symptoms at an early stage.

We found that patients were concentrated in top-tier hospitals in major cities and that hematologists and nephrologists in tertiary hospitals had the most opportunities to reach patients with AL amyloidosis. Discriminating AL amyloidosis from common diseases and other types of amyloidosis is difficult because AL amyloidosis is not only a rare disease, but is typically a multisystem disease, resulting in complex signs and symptoms with different systems involved [28,29]. As tertiary hospitals have abundant medical resources such as advanced diagnostic modalities and experienced physicians to support early recognition of rare diseases, early referral to a specialized center with expertise in management of AL amyloidosis is always recommended [29].

### Limitations

There are several limitations to this analysis. First, the data were collected from online doctor-patient communication platforms in China, but it is unclear whether the results of this study can be generalized to represent all AL amyloidosis patients even within China due to selection bias arising from the inclusion of only internet users. Second, the available data were constrained by what patients posted, possibly leading to inaccuracies and skewed data. Third, although we eliminated as many non-AL amyloidosis patients as possible, it is inevitable that we included other types of amyloidosis, as the patients were not clinically validated. Despite these limitations, this study offers a large sample of a rare disease and comprehensive insights that increase understanding of the real-world patient journey for AL amyloidosis in China.

### Conclusions

This study explored clinical characteristics and patient experience from symptom onset to diagnosis of AL amyloidosis in China. Patients experience referrals during their journey to an accurate diagnosis. Increasing awareness of the disease and early referral to a specialized center with expertise may reduce delayed diagnoses and improve patient management. For precise treatments, further evidence-based research on AL amyloidosis in China is required.

## Appendix

Multimedia Appendix 1 Supplementary tables and figures.

## Abbreviations

AL: light chain
BERT: bidirectional encoder representations from transformers
CRF: conditional random field
NLP: natural language processing

## Data Availability

The data set generated and analyzed during this study is available from the corresponding author on reasonable request.
