# Supplementary material for: Patient Journey Toward a Diagnosis of Light Chain Amyloidosis in a National Sample: Cross-Sectional Web-Based Study
Source: JMIR Form Res. 2023 Nov 2;7:e44420. doi: 10.2196/44420 (PMC10654903; doi:10.2196/44420)
Supplement: Multimedia Appendix 1 [file formative_v7i1e44420_app1.docx]

**02Supplementary Tables and Figures**

**Supplementary Table 1. Data source(patients)**

| Characteristic | Specific descriptor | Total number of posts |
| --- | --- | --- |
| Source of Information | Haodf.com  wy.guahao.com  Chunyuyisheng.com  ask.39.net  91160.com | 1,968 (89.29%)  118 (5.35%)  66 (2.99%)  34 (1.54%)  18 (0.82%) |
| Temporal distribution (year) | 2008  2009  2010  2011  2012  2013  2014  2015  2016  2017  2018  2019  2020  2021(Up to April) | 2  41  39  73  61  73  47  114  205  209  254  347  461  278 |
| Geographical distribution^▲^ | Jiangsu  Zhejiang  Beijing  Henan  Shandong  Anhui  Hebei  Hubei  Liaoning  Guangdong  Inner Mongolia  Shanxi  Jilin  Heilongjiang  Jiangxi  Guizhou  Hunan  Shaanxi  Shanghai  Sichuan  Gansu  Guangxi  Tianjin  Xinjiang  Hainan  Chongqing  Fujian  Qinghai  Yunnan  Ningxia  Tibet | 260 (11.80%)  181 (8.21%)  142 (6.44%)  142 (6.44%)  138 (6.26%)  131 (5.94%)  125 (5.67%)  125 (5.67%)  118 (5.35%)  115 (5.22%)  79 (3.58%)  76 (3.45%)  69 (3.13%)  59 (2.68%)  59 (2.68%)  55 (2.50%)  54 (2.45%)  42 (1.91%)  38 (1.72%)  35 (1.59%)  31 (1.41%)  31 (1.41%)  21 (0.95%)  17 (0.77%)  17 (0.77%)  14 (0.64%)  10 (0.45%)  10 (0.45%)  10 (0.45%)  0 (0)  0 (0) |
|  |  |  |

^▲^Data from Hong Kong, Macao and Taiwan was not available.

**Supplementary Table 2. Patients Characteristics**

| Characteristic | Mentioned posts (ratio) |
| --- | --- |
| Gender, n = 2204, n(%)  Female  Male | 920 (41.74%)  1,284 (58.26%) |
| Age, n = 196, Median(range), year | 57 (21 - 87) |
| specific symptoms related keywords, n = 2,204, n (%)  Kidney-related  Heart-related  Liver-related  Stomach-related  Lung-related  Intestines-related  Nervous system-related | 1,459 (66.20%)  833 (37.79%)  491 (22.28%)  368 (16.70%)  306 (13.88%)  280 (12.70%)  225 (10.21%) |
| numbers of organ-related symptoms, n = 2,204, n (%)  1  2  3  4  5  6  7 | 702 (31.85%)  619 (28.09%)  395 (17.92%)  226 (10.25%)  128 (5.81%)  72 (3.27%)  62 (2.81%) |

**Supplementary Figure 1.** **Treatment in patients with AL-amyloidosis**

**A**

**A. Overview of treatment mentioned by doctors (n = 1,688)**

、

**B**

**B. Overview of regimens in first-line and second-line treatments. (n = 164)**

V: Bortezomib, C: Cyclophosphamide, d: Dexamethasone, D: Daratumumab, I: Ixazomib, M: Melphalan, R: Lenalidomide, T: Thalidomide, VD: Bortezomib & Daratumumab
